# Targeting Autoinducer‑2 Quorum Sensing: Novel Inhibitors Attenuate Virulence in *Staphylococcus aureus* and *Pseudomonas aeruginosa*

**DOI:** 10.1021/acsomega.5c08721

**Published:** 2026-04-02

**Authors:** Angelica Pellegrini, Giorgio Milli, Elisa Bellan Menegussi, Simona Viglio, Pietro Cinaglia, Roberta Listro, Pasquale Linciano, Simona Collina, Giampiero Pietrocola

**Affiliations:** † Department of Molecular Medicine, Biochemistry Unit, 19001 University of Pavia, Viale Taramelli 3/b, 27100 Pavia, Italy; ‡ Department of Drug Sciences, University of Pavia, Viale Taramelli 12, 27100 Pavia, Italy; § Department of Health Sciences, University Magna Graecia, Italy; Data Analytics Research Center, University Magna Graecia, 88100 Catanzaro, Italy

## Abstract

Antibiotic overuse has driven the development of bacterial drug resistance, highlighting the urgent need for new antimicrobial strategies. Quorum sensing, particularly via autoinducer-2 (AI-2) signal molecules, is a bacterial communication system involved in the development of drug resistance. Our recent work identified AI-2 inhibitors that disrupt biofilm formation in *Staphylococcus aureus* and *Pseudomonas aeruginosa*. Here, we investigated the efficacy of the three most promising compounds in modulating bacterial virulence. The compounds strongly impaired the bacterial ability to adhere to human epithelial cells. ELISA-based assays and Western blot analyses revealed the efficacy of the compounds in controlling *S. aureus* virulence by decreasing the levels of bacterial adhesins, α-hemolysin, and protein SpA. In a co-culture with *S. aureus*, colorimetric assays revealed the compound efficacy in decreasing *P. aeruginosa* pyocyanin and elastase production in a dose-dependent manner. Importantly, no cytotoxicity was observed both in vitro (A549 cells) and in vivo (*Galleria mellonella* model). These results support the potential of our compounds to modulate virulence factor expression consistent with quorum-sensing disruption as promising candidates for the treatment of multidrug-resistant infections.

## Graphical abstract

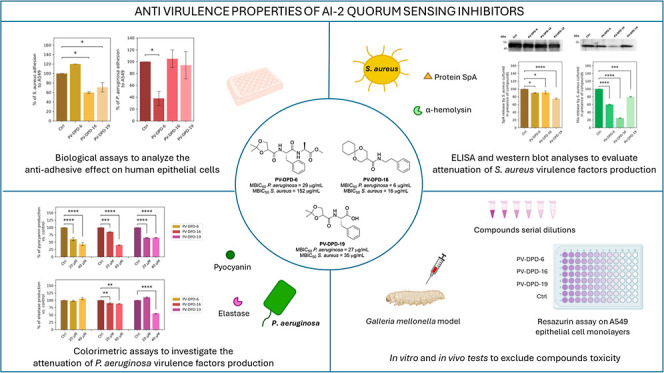

## 1 Introduction

Infections caused by multidrug-resistant (MDR) bacteria represent an escalating and pressing global public health threat. *Staphylococcus aureus* and *Pseudomonas aeruginosa*—both members of the ESKAPE group—are among the most clinically relevant pathogens, standing out due to their combination of high virulence potential and remarkable ability to develop MDR.^1,2^ The World Health Organization has classified methicillin-resistant *S. aureus* and carbapenem-resistant *P. aeruginosa* as high-priority bacterial pathogens, requiring tailored intervention strategies to fight bacterial resistance to antibiotics.^3^

*S. aureus* is a Gram-positive asymptomatic colonizer of the human population, which can turn into an opportunistic pathogen, causing a broad spectrum of diseases, ranging from mild soft tissue and skin infections to pneumonia and endocarditis.^4^ Its pathogenicity is largely attributed to a diverse arsenal of virulence factors, notably, cell-wall-anchored (CWA) adhesins, which facilitate adherence to host tissues and surfaces. These comprise different families, such as the microbial surface components recognizing adhesive matrix molecules (MSCRAMMs), including the multivalent fibronectin binding protein FnBPA and B, the clumping factor ClfA and B, the serine aspartate repeat Sdr family, and SraP.^5^ Additional key virulence factors from *S. aureus* include hemolysins, which contribute to host cell lysis and impair the immune responses from host cells.^6,7^ This entire array of virulence factors contributes collectively to increase the high pathogenic potential of this pathogen.^8^

*P. aeruginosa*, an opportunistic Gram-negative pathogen, primarily infects immunocompromised individuals, causing acute to chronic infections, often difficult to eradicate due to the high mutation rate that confers the bacterium an intrinsic resistance to antibiotics.^9^ This bacterium secretes a wide range of virulence factors that allow bacterial adaptation to different host niches, including pyocyanin, a cytotoxic pigment able to damage host tissue,^10^ and elastase, a protease that promotes tissue invasion and biofilm formation.^11^

Co-infection of *S. aureus* and *P. aeruginosa* in the ear, nose, and throat (ENT) presents a complex clinical challenge due to the interactions between these pathogens and their impact on disease severity and treatment outcomes. These bacteria are frequently found together in polymicrobial infections, such as chronic otitis and cystic fibrosis (CF), where they can exacerbate the condition and complicate treatment strategies,^12^ while in chronic rhinosinusitis, biofilm formation by these bacteria is associated with poorer surgical outcomes and persistent infections.^13^ In CF, *S. aureus* is usually among the first bacterial colonizers of lungs in children,^13^ while *P. aeruginosa* overtakes *S. aureus* at later stages of infection. *P. aeruginosa* can outcompete *S. aureus*, potentially leading to more aggressive infections,^14^ but in CF, these pathogens often coexist, with *P. aeruginosa* typically becoming more dominant as patients age. However, *S. aureus* remains significant, and its coinfection is linked to worse clinical outcomes.^15,16^ Thus, in CF patients, coinfection correlates with worsened clinical outcomes, including reduced lung function, increased exacerbation rates, and higher hospitalization frequency.^15–17^ Similarly, in ENT infections, biofilm formation by both pathogens is a key factor driving chronicity, antibiotic resistance, and treatment failure. The interactions between *S. aureus* and *P. aeruginosa* can range from competitive to mutualistic, often resulting in enhanced virulence and more severe infections. Interestingly, coculturing these bacteria has been shown to enhance *P. aeruginosa* production of pyocyanin and elastase compared to monoculture conditions, while promoting structured biofilm coformation, thereby further increasing their collective resilience to antibiotics.^18^

Biofilm formation is a critical step in the persistence of bacterial infections, as these complex microbial communities shield bacteria from host immune responses and drastically reduce antibiotic efficacy.^19^ In recent years, several natural and synthetic compounds have been explored for their antibiofilm properties;^20^ however, their effects on virulence modulation and pathogen–pathogen interactions during coinfection remain poorly understood. Biofilm production and a great number of virulence factors are regulated by a complex coordination of signals governed by quorum sensing (QS). QS is that phenomenon whereby the cell population is able to sense the accumulation of specific, diffusible signal molecules, or “autoinducers” (AI), and understand that the minimal number, or “quorum,” of bacteria has been achieved for a certain response to be activated.^21^ Bacteria display several AI systems. Gram-negatives commonly use LuxR-type receptors, cytoplasmic transcription factors that detect acyl-homoserine lactones (AHLs) produced by their partner LuxI-type synthases.^22^ Gram-positive bacteria adopt nonmembrane diffusible peptide-based signals, or autoinducing peptides (AIPs).^23^ Moreover, both Gram-negative and -positive bacteria display 4,5-dihydroxy-2,3-pentonedione-derived (DPD) signal molecules, called autoinducer-2 (AI-2).^24,25^ AI-2 molecules are usually sensed through two-component membrane-bound histidine kinases that use phosphorylation to signal cytoplasmic transcription factors.^26^ In this context, the *lsrACDBFGE* (*lsr*, LuxS regulated) operon encodes an ATP-binding cassette transporter (ABC transporter) that internalizes AI-2.^27^

Among the major regulators of QS in *S. aureus*, the accessory gene regulator (*agr*) is a system activated via AIP.^28^ It plays a central role in modulating the expression of several virulence factors, such as the surface-associated proteins ClfA, ClfB, and FnBPs, and the secretion of virulence factors during different infection stages, such as α-hemolysin (Hla) and protein A (SpA).^29–31^ The LuxS/AI-2 system, the unique QS system shared by both Gram-positive and Gram-negative bacteria, is the major regulator governing staphylococcal biofilm formation.^32^

In *P. aeruginosa*, QS involves a hierarchical system comprising, among others, the Las system, which controls the expression of elastase and biofilm production, and Rhl and Pqs circuits, which regulate pyocyanin and biofilm production.^33,34^ Interestingly, *P. aeruginosa* is not able to produce AI-2, as it does not encode LuxS. Nonetheless, it is able to sense AI-2 produced by other bacterial species, through unique receptors, and regulate its gene expression accordingly.^35,36^

While conventional antibiotic therapies primarily aim to kill or inhibit bacterial growth, alternative approaches targeting QS-regulated virulence pathways represent a new viable strategy to deal with AMR. Since most virulence determinants are not essential for bacterial viability, their inhibition is less likely to exert selective pressure for resistance. Antivirulence strategies based on QS interference thus represent a promising nonbactericidal approach to disarm pathogens without promoting resistance. In this context, small molecules that selectively interfere with QS signaling have emerged as attractive candidates for the management of polymicrobial and drug-resistant infections.

Accordingly, in our previous studies,^37^ ^,^ ^38^ we adopted a ligand-based design strategy starting from the chemical structure of DPD, the native AI-2 quorum-sensing autoinducer (Figure 1A), to develop a focused series of DPD-inspired small molecules acting as selective inhibitors of AI-2-mediated quorum sensing (QSIs). From this series, three compounds, namely, PV-DPD-6, PV-DPD-16, and PV-DPD-19 (Figure 1B), emerged as the most potent and promising hits. These molecules markedly impaired biofilm formation in both *P. aeruginosa* and *S. aureus*, displaying MBIC_50_ values in the low μg/mL range, while notably lacking direct antibacterial activity, consistent with a nonbactericidal antivirulence mechanism.

**Figure 1:**
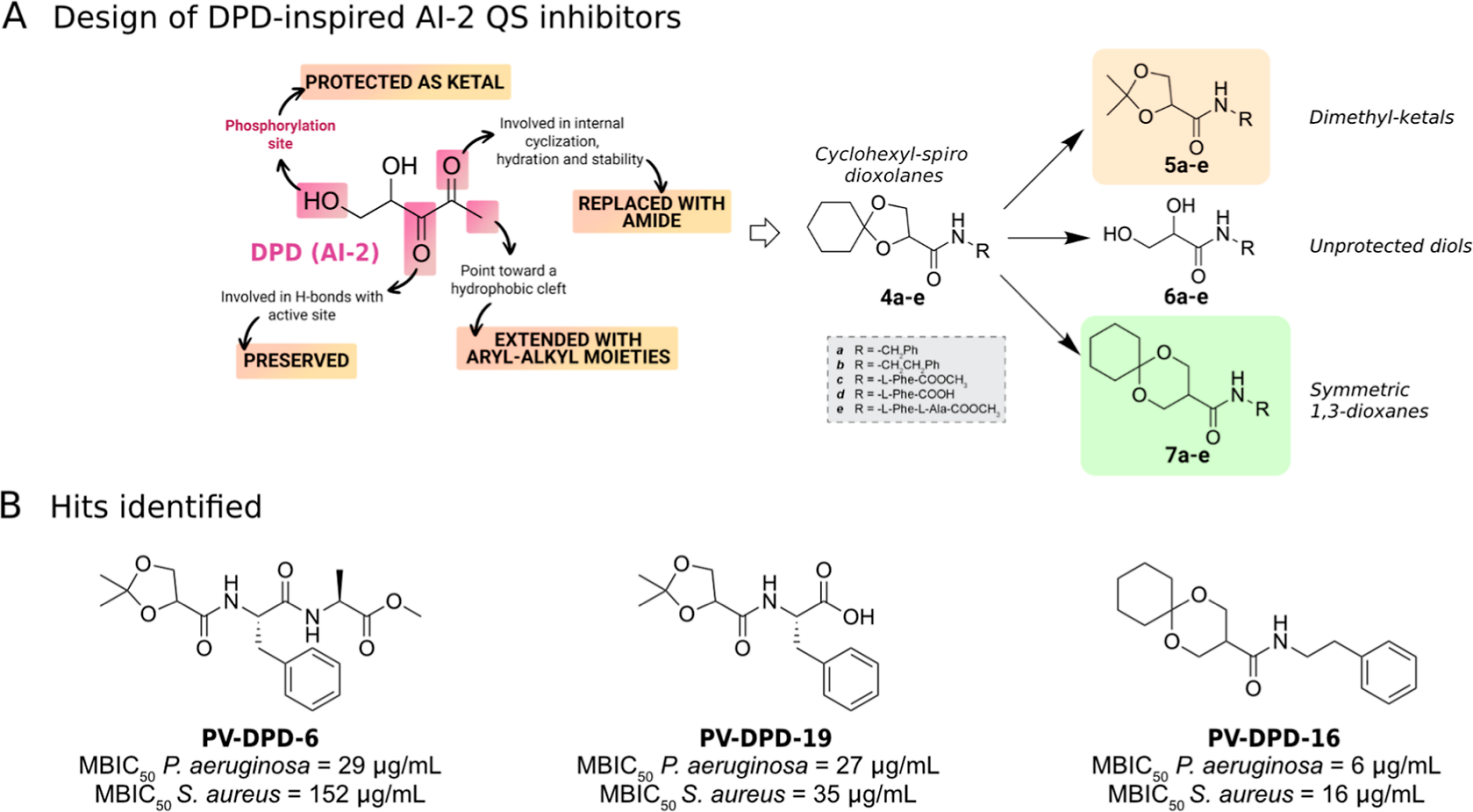
(A) Ligand-based strategy starting from the native AI-2 autoinducer DPD for the design of structurally inspired analogues.^38^ (B) Chemical structures and MBIC_50_ values (Minimum Biofilm Inhibitory Concentration, i.e., the lowest concentration of a compound required to inhibit 50% of biofilm formation relative to an untreated control) against *P. aeruginosa* and *S. aureus* for the most promising hit compounds PV-DPD-6, PV-DPD-16, and PV-DPD-19.^38^

In the present study, we report a deeper functional characterization of these three QSIs with the aim of validating the antivirulence potential and providing novel experimental evidence to support their development in such alternative strategies to managing chronic and difficult-to-treat infections. We evaluated their ability to impair key virulence traits, including surface adhesins, α-hemolysin, and protein A in *S. aureus*, as well as pyocyanin and elastase production in *P. aeruginosa*, under both monoculture and coculture conditions. Given the central role of AI-2 in regulating adhesion-related factors in *S. aureus*, we specifically investigated the impact of these compounds on the expression of MSCRAMM adhesins. Additionally, we assessed bacterial adhesion to epithelial cells and compound toxicity in vitro and in vivo using the *G. mellonella* larvae model.

## 2 Results and Discussion

### 2.1 Effect of Compounds on *Pseudomonas aeruginosa* and *Staphylococcus aureus* Adhesion to Human Pulmonary Epithelial Cells

Bacterial adhesion to host tissues is a critical initial step in the establishment of successful infections as it facilitates tissue colonization and eventual subsequent invasion. We evaluated the antiadhesive properties of our selected compounds through in vitro adhesion assays using human lung epithelial A549 cells. Bacterial cultures were incubated with 100 μM of each compound overnight at 37 °C. Notably, *S. aureus* overnight exposure to compounds PV-DPD-16 and PV-DPD-19 led to a significant decrease in bacterial adherence to epithelial cells, approximately 40% and 30%, respectively, compared to the untreated control (Figure 2). This result is particularly noteworthy, as it suggests a direct effect on the expression or function of bacterial surface adhesins, which are crucial for the initial stages of host interaction and biofilm formation. In contrast, compound PV-DPD-6 did not affect *S. aureus* adhesion.

**Figure 2:**
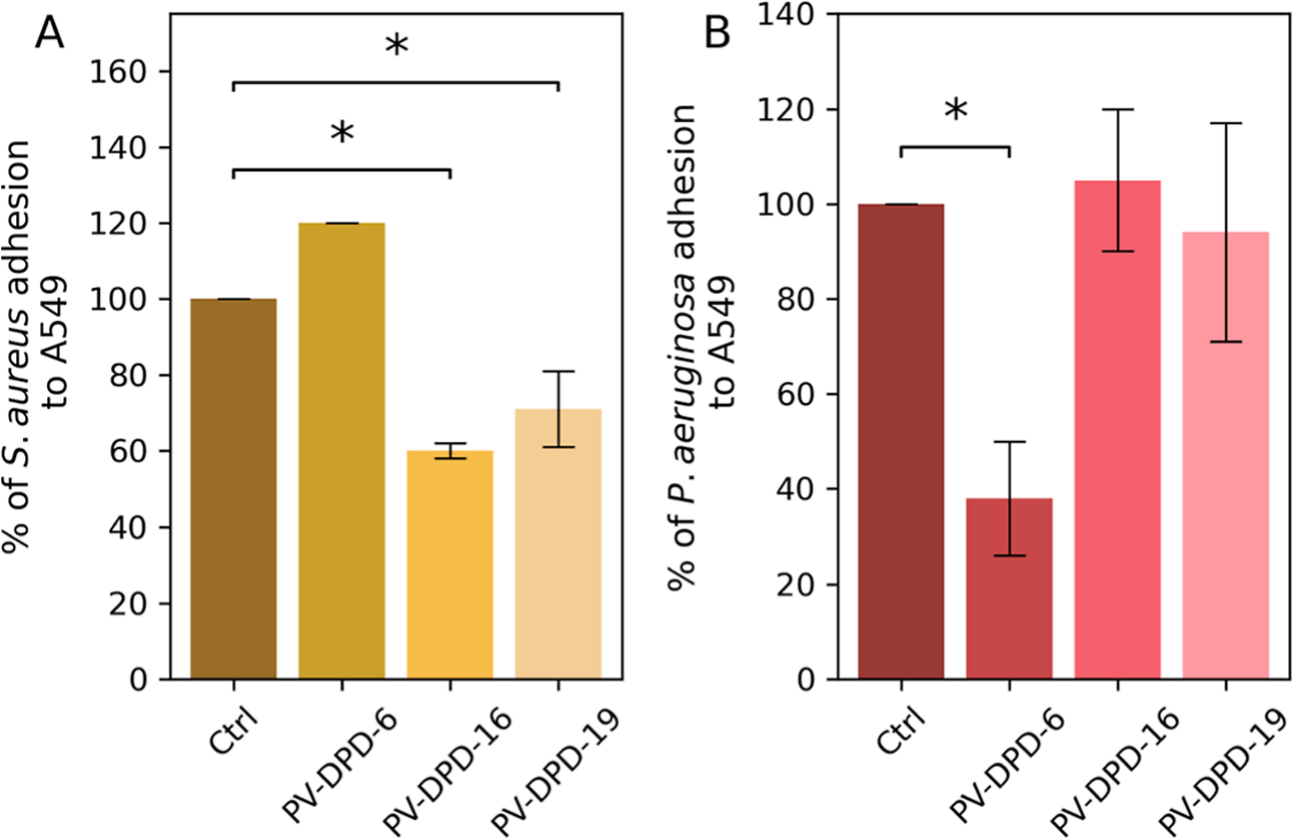
Adhesion to epithelial cells by *S. aureus* and *P. aeruginosa* cultured in the presence or absence of compounds. (A) *S. aureus* adhesion impaired by compounds PV-DPD-16 and PV-DPD-19. (B) *P. aeruginosa* adhesion impaired by compound PV-DPD-6. Cell adhesion was assessed using monolayers of the A549 immortalized cell line. The adhesion percentage for each strain was calculated relative to the initial inoculum and normalized to that of the untreated strain, which was defined as 100%. Data are presented as the mean ± SD from three independent experiments, each conducted in duplicate. Statistically significant differences are reported (*p < 0.05, one-way ANOVA).

Conversely, *P. aeruginosa* adhesion was strongly impaired following treatment with PV-DPD-6, whereas compounds PV-DPD-16 and PV-DPD-19 had no detectable effect on adherence. These findings align with previous biofilm inhibition studies using the same compounds, which showed similar patterns of activity,^38^ and suggest a clear species-specific response to the tested AI-2 inhibitors, suggesting that the mechanisms regulating adhesion in *S. aureus* and *P. aeruginosa* may be differentially influenced by QS interference.

Interestingly, this selective interference could be advantageous in polymicrobial infection contexts, where targeted suppression of one pathogen may be achieved without necessarily affecting commensal or nontarget species. Moreover, given the nonbactericidal nature of these compounds, they are less likely to induce resistance and may preserve host microbiota integrity. Together, these results highlight the potential of specific compounds to selectively interfere with early-stage host colonization by *S. aureus* and *P. aeruginosa*, supporting their further investigation as antivirulence agents.

### 2.2 Effect of Compounds on Virulence Factors of *S. aureus*

To assess the impact of the tested compounds on the expression of *S. aureus* virulence factors, we investigated the expression of multiple MSCRAMM adhesins in the SH1000 Δ*spa* strain cultured in TSB in the presence or absence of each compound (100 μM). This strain, a derivative of the SH1000 strain deleted for the SpA protein, was used to avoid nonspecific binding during the assay, as SpA can bind to the Fc region of IgG and potentially interfere with signal detection.^39^ Following overnight incubation, bacterial cells were immobilized onto a microtiter plate, and protein expression was quantified by an ELISA-based in vitro assay, using specific IgG antibodies against selected MSCRAMMs. As shown in Figure 3, compound PV-DPD-6 significantly reduced Clumping factor A (ClfA) expression and displayed no notable effect on the other adhesins. Compound PV-DPD-16 impaired both ClfA and Fibronectin-binding protein A (FnBPA) expression. The most pronounced activity was observed with compound PV-DPD-19, which significantly impaired the expression of several MSCRAMMs, including serine-aspartate repeat protein C (SdrC), bone sialoprotein-binding protein (Bbp), ClfA, clumping factor B (ClfB), FnBPA, and fibronectin binding protein B (FnBPB).

**Figure 3:**
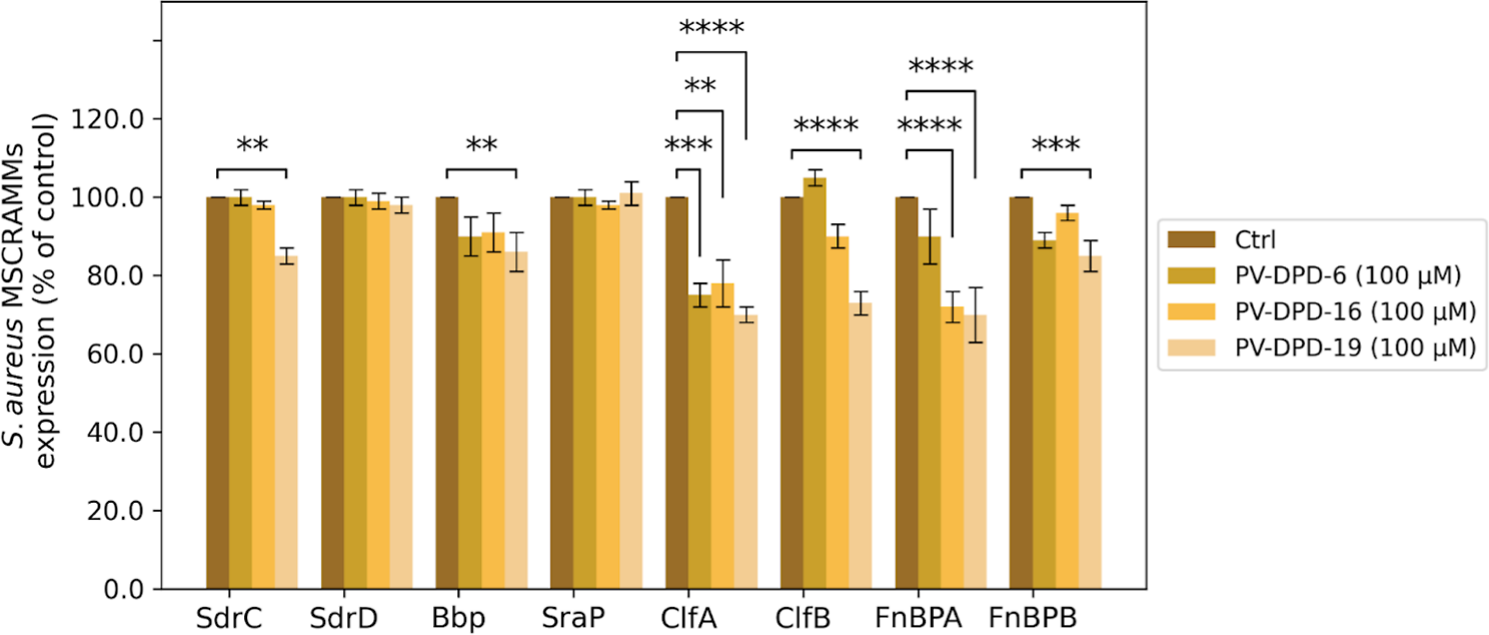
ELISA detection of adhesins expressed on *S. aureus* cells cultured in the presence or absence of compounds. Cells were grown in the presence of 100 μM of compounds PV-DPD-6, PV-DPD-16, or PV-DPD-19. Protein detection is represented as a percentage of the control (untreated strain), set at 100%. Data are presented as the mean ± SD from three independent experiments, each carried out in duplicate. Statistical significance is indicated (**p < 0.01, ***p < 0.001, ****p < 0.0001) (one-way ANOVA).

The observed selective inhibition of bacterial adhesins suggests a possible interference of the tested compounds with global QS networks, which coordinate the expression of bacterial pathogenic traits in response to cell density and environmental signals. In *S. aureus*, the *agr* system plays a central role in modulating the switch between surface-associated MSCRAMM proteins, such as ClfA, ClfB, and FnBPs, according to different stages of bacterial growth.^28^ The simultaneous downregulation of multiple adhesins investigated in our study, particularly by compounds PV-DPD-16 and PV-DPD-19, suggests that these compounds might interfere, directly or indirectly, with the *agr* system.

In addition to evaluating the expression of cell-surface adhesins, we also tested whether the compounds could affect the production of *S. aureus* virulence factors secreted into the culture medium. We conducted Western blot analyses on bacterial supernatants from cells cultured overnight in the presence or absence of compounds at a concentration of 100 μM. Quantification of Spa protein and Hla hemolysin production by the SH1000 strain was performed through densitometric evaluation of immunoblot signals at the protein level. This analysis demonstrated that all three compounds were able to impair both virulence factor release (Figure 4). In particular, compounds PV-DPD-6 and PV-DPD-16 led to a 10% decrease of SpA detected in the medium, while compound PV-DPD-19 was able to reduce about 30% the release of SpA protein. Concerning the hemolysin Hla, we obtained more heterogeneous results, with a 40%, 70%, and 90% decrease in the presence of compounds PV-DPD-6, PV-DPD-16, and PV-DPD-19, respectively.

**Figure 4:**
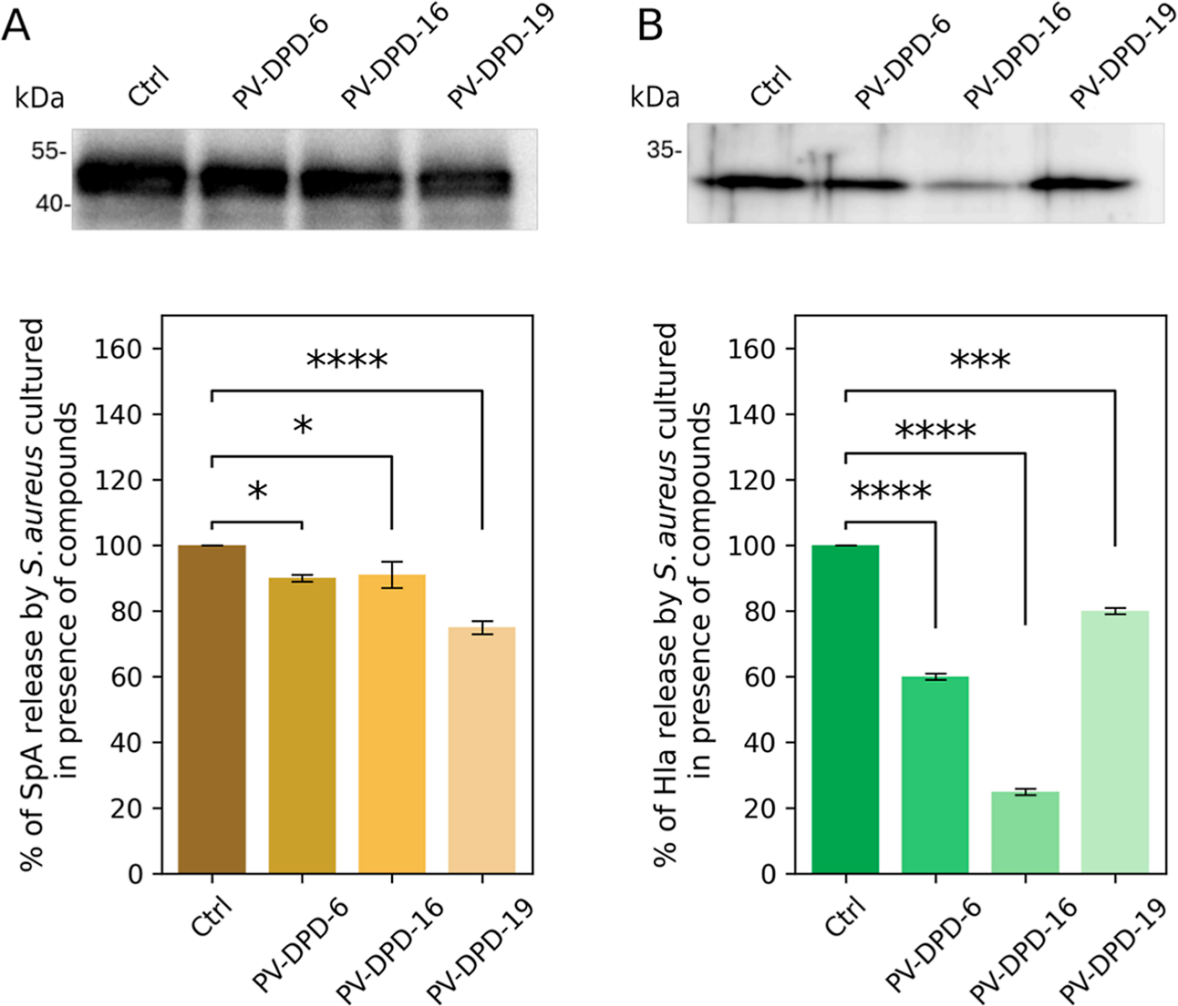
Production of protein A and α-toxin in the presence of compounds assayed by Western blot in SH1000. Cells were cultured in the presence of 100 μM PV-DPD-6, PV-DPD-16, or PV-DPD-19. Bacterial supernatants were separated by 15% SDS-PAGE and subsequently transferred to a PVDF membrane. Protein expression was detected using anti-SpA (A) and anti-Hla (B) antibodies. Data are expressed as a percentage of the untreated control strain, which was defined as 100%. The presented data are representative of three independent experiments. Statistically significant differences are indicated. *p < 0.05, ***p < 0.001, ****p < 0.0001 (one-way ANOVA).

Interestingly, despite the regulation of adhesin expression, the *agr* system plays a role in the regulation of expression of secreted virulence factors as well, such as the Hla α-hemolysin and the SpA protein.^29^ The observed downregulation of *hla* and *spa* expression in response to the compounds under investigation provides further support for the hypothesis that these agents may interfere with *agr*-mediated regulatory pathways. This interference could occur either through direct modulation of the *agr* system or via upstream regulatory elements, such as SarA or Rot, which are known to influence *agr* expression and activity. Moreover, previous studies conducted by Yu et al. have demonstrated a cooperative interaction between the *agr*-mediated QS system and the LuxS/AI-2 signaling pathway, resulting in a cumulative effect on biofilm formation.^32^ Considering the nature of our compounds as directed toward an AI-2 QS system, we could plausibly hypothesize that our compounds exert their effects indirectly on the *agr* system by modulating the AI-2-dependent QS signaling. Further investigations are required to delineate the precise molecular mechanisms underlying these observations.

### 2.3 Effect of Compounds on *S. aureus* AI-2 Production

The ability of PV-DPD-6, PV-DPD-16, and PV-DPD-19 to inhibit *S. aureus* AI-2 production was investigated through the *Vibrio harveyi* bioluminescence assay. To assess AI-2 activity, the *S. aureus* SH1000 strain was cultured overnight in the presence or absence of compounds PV-DPD-6, PV-DPD-16, or PV-DPD-19 (2 mM). The AI-2 reporter *V. harveyi* strain BAA-1117 was incubated in the presence or absence of cell-free supernatants (CFS) derived from each overnight culture, and luminescence levels were quantified after 8 h of incubation. AI-2 signaling activity is indicated as the percentage of *V. harveyi* bioluminescence (normalized on cell growth) incubated in the presence of CFS from *S. aureus* cultured in the presence of each compound, relative to untreated control (CFS from *S. aureus* incubated without compounds), set as 100%. As reported in Figure 5, both PV-DPD-16 and PV-DPD-19 compounds induced a significant reduction in luminescence, indicating decreased levels of AI-2 compared to CFS from an untreated *S. aureus* culture. This result supports the role of these two compounds as QS inhibitors targeting AI-2-mediated signaling in *S. aureus*. In contrast, the compound PV-DPD-6 caused a slight yet not statistically significant decrease in luminescence, suggesting a limited effect on AI-2 production or activity under the tested conditions. Overall, these findings are consistent with our results on the effects of the compounds on *S. aureus* virulence and further support their specificity in modulating virulence traits through the AI-2-dependent QS pathway. Nonetheless, further experiments will be required to unequivocally demonstrate direct AI-2 antagonism by our compounds.

**Figure 5:**
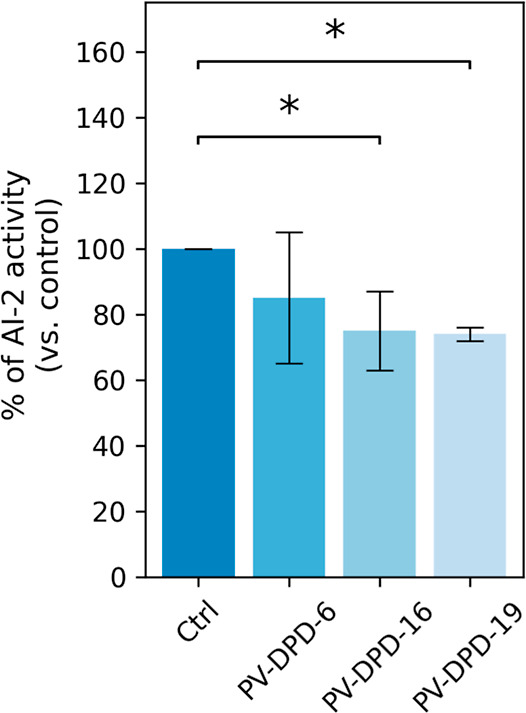
Effect of compounds on *S. aureus* AI-2 production assessed by a *V. harveyi* bioluminescence assay. *V. harveyi* BAA-1117 cells were incubated in the presence of CFS from *S. aureus* cultures grown in the presence or absence of 2 mM of compounds PV-DPD-6, PV-DPD-16, or PV-DPD-19, 8 h, 30 °C, shaking at 100 rpm. AI-2 activity values are represented as a percentage of control (no compound added in medium), set at 100%. The presented data are representative of three independent experiments. Statistically significant differences are indicated. *p < 0.05 (one-way ANOVA).

### 2.4 Effect of Compounds on Virulence Factors of *P. aeruginosa*

The effect of the compounds on virulence factor production in *P. aeruginosa* PAO1 was evaluated. As previously published, *P. aeruginosa* production of virulence factors is enhanced when the bacterium is cultured in the presence of *S. aureus*.^18,40^ Therefore, we quantitatively analyzed the effects of our compounds on the production of pyocyanin and elastase in *P. aeruginosa* PAO1 during coculture with the *S. aureus* SH1000 strain. Both virulence factors were quantified using a colorimetric assay.

As displayed in Figure 6, the production of pyocyanin significantly decreased in a dose-dependent manner when *P. aeruginosa* was cultured in the presence of each compound tested (separately). In particular, compounds PV-DPD-6 and PV-DPD-16 reduced the production of pyocyanin up to ∼60% when tested at a concentration of 40 μM, compared with the untreated control, suggesting a strong inhibitory effect on the phenazine biosynthetic pathway (Figure 6A). These results confirm that the compounds inhibit the production of *P. aeruginosa* virulence factors, showing greater efficacy than the previously reported compound Str7410, which displayed a 22% and 25% inhibition of pyocyanin and elastase production, respectively, when tested at a concentration of 40 μM.^18^ Pyocyanin is a secondary redox-active metabolite whose expression is controlled by *las* and *rhl* QS systems, the Pqs and AI-2 system, which display a role in the integration of environmental signals and interactions among species to coordinate virulence.^41,42^ The suppression of pyocyanin observed when *P. aeruginosa* is cocultured with *S. aureus* in the presence of these compounds implies interference with QS-mediated regulation that could potentially target the above-mentioned QS receptors.

**Figure 6:**
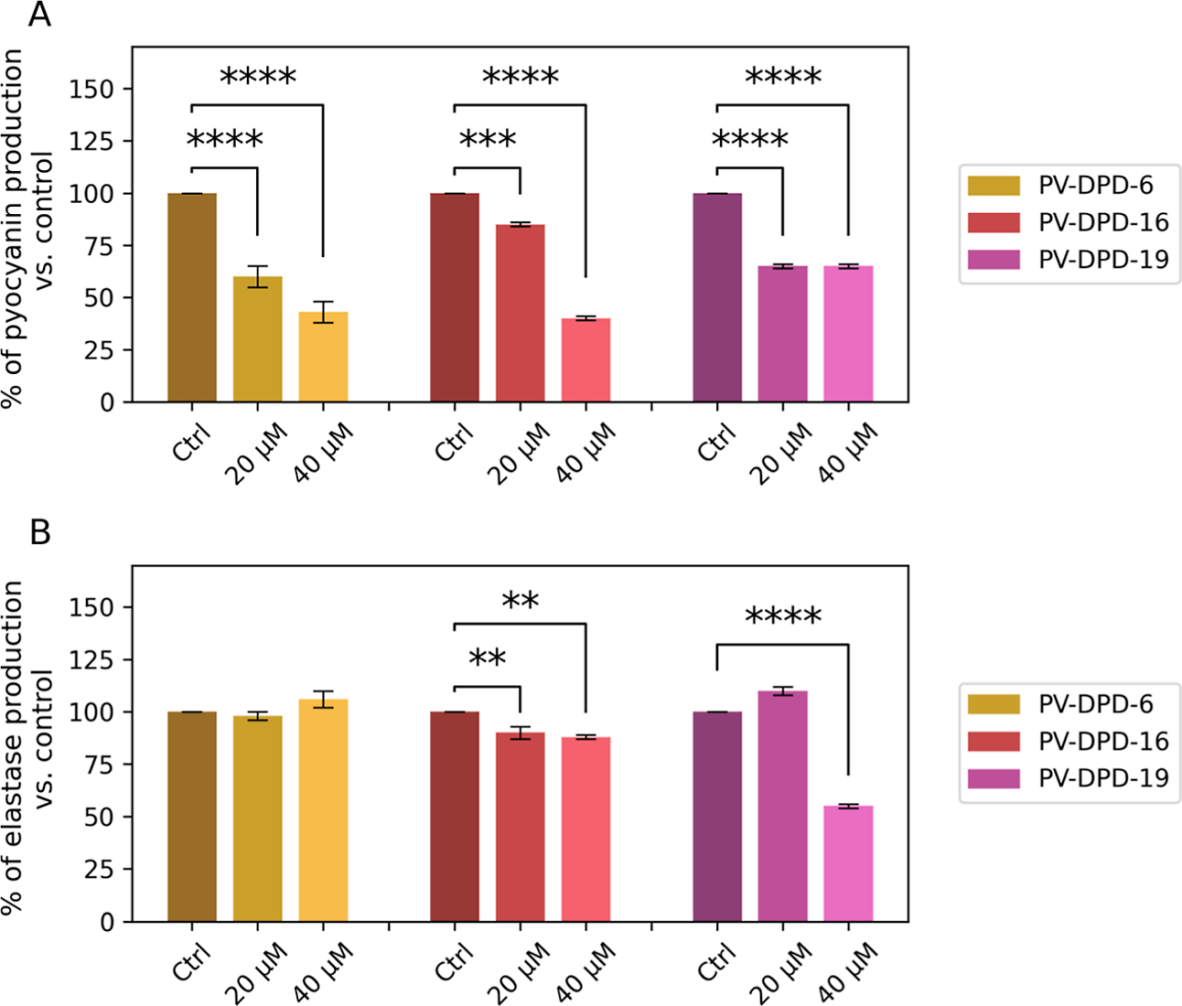
Production of pyocyanin (A) and elastase (B) by *P. aeruginosa*in coculture with *S. aureus*in the presence or absence of compounds. Cells were cultured in the presence of 20 and 40 μM of compounds PV-DPD-6, PV-DPD-16, or PV-DPD-19. Pyocyanin levels were detected by extracting pyocyanin from the bacterial supernatant through chloroform and HCl and then measuring the absorbance at 520 nm. Elastase was measured through the Elastin–Congo red assay, with absorbance recorded at 495 nm. Results are expressed as a percentage of the untreated control, which was defined as 100%. Data shown are derived from three independent experiments. Statistically significant differences are reported (**p < 0.01, ***p < 0.001, ****p < 0.0001) (two-way ANOVA).

Different from what was observed for pyocyanin, the compound PV-DPD-6 did not display any effect on the elastase production under the concentrations tested (Figure 6B). Concerning compounds PV-DPD-16 and PV-DPD-19, the elastase production was repressed in a dose-dependent manner, with compound PV-DPD-19 displaying the major effect. At a concentration of PV-DPD-19 40 μM, elastase production was inhibited by around 50%, compared to no treatment. As elastase expression is regulated mainly by the Las QS system through the LasR transcriptional activator, this system could be targeted by our compounds. Nonetheless, the differential activity of compounds for the two virulence factors suggests distinct modes of action among them. This result could possibly reflect varying affinities of our compounds under investigation for specific regulatory targets or QS circuits. Notably, elastase and pyocyanin are partially coregulated by the *las* system, but they can also be differentially expressed, on the basis of additional regulatory inputs.^43^

Several studies focused on the development of inhibitors that act on QS systems mediated by AHL signaling molecules in *P. aeruginosa*.^44^ In light of the ability of *P. aeruginosa* to perceive AI-2 signaling molecules synthesized by other bacteria, more recent research has focused on the study of inhibitors able to act at the AI-2 level.^18^ Our compounds have been recently characterized as able to interfere specifically with the AI-2 QS system.^38^ The plethora of virulence factors that emerged as affected by these compounds, together with the complex, intricate regulatory pathways governing their expression, makes it challenging to pinpoint a specific molecular target on which the compounds act, thereby complicating the elucidation of a clear and direct mechanism of action.

### 2.5 Evaluation of In Vitro and In Vivo Cytotoxicity of Compounds

To evaluate the potential toxicity of the compounds on host cells, both in vitro and in vivo toxicity assays were performed. For the in vitro analysis, compounds were tested on the human alveolar basal epithelial carcinoma (A549) cell line using the resazurin test. The test is based on the reduction of resazurin, a blue non-fluorescent dye, to resorufin, a pink, fluorescent product, by metabolically active cells. Therefore, resazurin is a cell-permeable indicator that can be used to monitor cellular viability.^45^ As the targets of these compounds are bacteria that infect the respiratory tract, testing the compounds’ degree of toxicity on the pulmonary A549 cell line gives us a higher accountability of results in correlation with future investigations. Both compounds were tested in concentrations starting from 3 up to 200 μM. Cell viability was maintained at 100% for all compounds at all concentrations tested, confirming their lack of cytotoxicity for the cell line tested (Figure 7).

**Figure 7:**
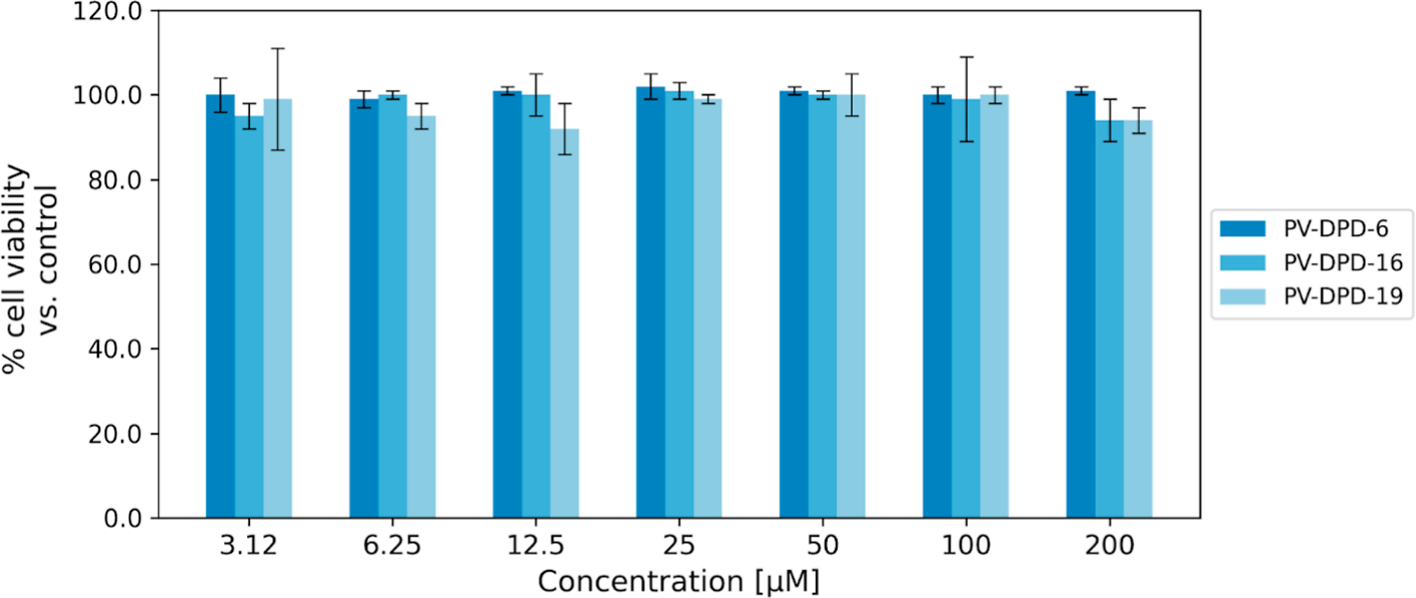
Evaluation of cytotoxicity of compounds for A549 cells. Cell monolayers were incubated in the presence of increasing concentrations of compounds PV-DPD-6, PV-DPD-16, or PV-DPD-19 (up to 200 μM). Cell viability was determined using the resazurin assay and was quantified by reading resorufin fluorescence (ex: 520 nm, em: 580 nm). Values are reported as a percentage of the untreated control, which was set as 100%. The reported data represent three independent experiments. Statistical analyses were conducted using the two-way ANOVA test.

For the in vivo tests, compound toxicity was assessed using the *Galleria mellonella* larvae model (Figure 8). *G. mellonella* larvae are classified as nonanimal technologies, yet they display elements of mammalian complexity; therefore, they are widely accepted as an ethical alternative for research.^46^ Compounds were injected at increasing concentrations (1-100-200 μM), and larval survival was monitored for 72 h. No significant differences in survival were observed between the treated and untreated groups, indicating no detectable acute toxicity under the conditions tested. The results are presented as the mean percentage survival of *G. mellonella* larvae compared to the untreated control, as a function of the test compounds administered dosage (Table 1). All experiments were performed in triplicate.

**Figure 8:**
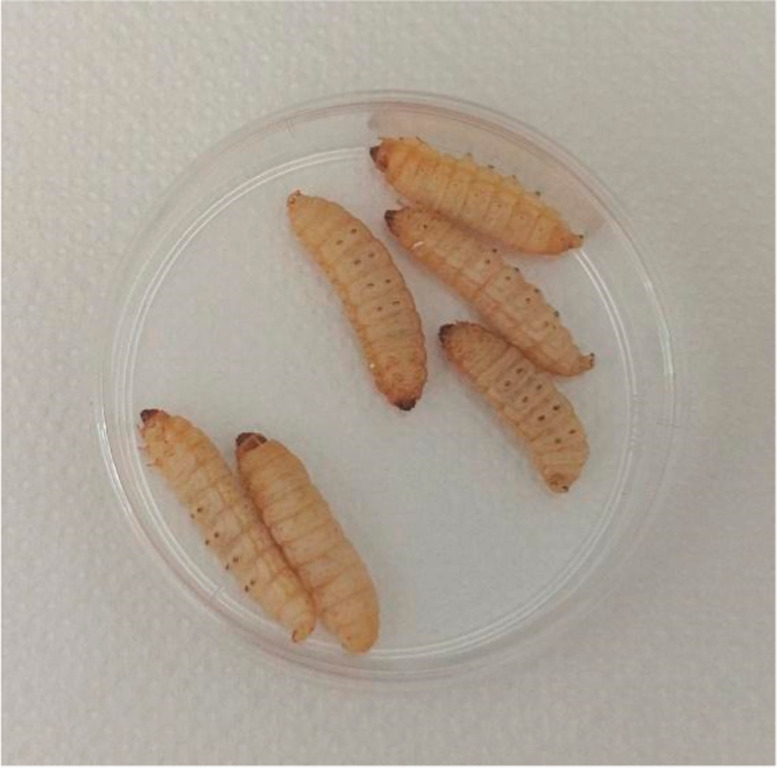
*Galleria mellonella* larva model.

**Table 1:** Survival of *G. mellonella* Larvae (Expressed as %)

compound	dosage concentration (μM)	G. mellonella survival (%)		
		24 h	48 h	72 h
PV-DPD-6	1	100	98	98
	100	100	100	98
	200	100	100	98
PV-DPD-16	1	100	100	100
	100	100	100	97
	200	100	100	100
PV-DPD-19	1	100	100	98
	100	100	97	97
	200	100	100	98
PBS	100	98	98	
DMSO	100	100	98	

## 3 Conclusions

Bacterial drug resistance is increasing due to the abuse of antibiotics. In multibacterial infections, bacterial pathogens communicate via QS systems to promote drug resistance and pathogenicity.^47^ *P. aeruginosa* and *S. aureus* are two bacterial pathogens causing a wide array of diseases, which can also coexist, such as during infections in cystic fibrosis patients. In recent years, studies have been focusing on the development of QS inhibitor molecules as an alternative strategy to overcome bacterial resistance.^18^

In our recent work, we identified several compounds, mimicking the DPD molecule governing the AI-2 driven QS, as able to interfere with the production of biofilm by *P. aeruginosa* and *S. aureus*.^38^ Given the growing interest in antivirulence strategies as an alternative to traditional antibiotics, we dedicated this research to a better characterization of the three best candidates, in terms of ability to reduce bacterial virulence. We found that the production of *S. aureus* adhesins and secreted proteins, such as SpA and Hla, was negatively affected when the bacterium was cultured in the presence of compounds. This result could possibly explain the impaired ability of the bacterium to adhere to human epithelial cells as well.

*P. aeruginosa* is able to sense AI-2 signal molecules produced by other bacteria and coordinate the expression of its virulence factors accordingly.^48^ *P. aeruginosa* virulence was inhibited by compounds, both in the bacterium alone, concerning its ability to adhere to human cells, and when in coinfection with *S. aureus*, where pyocyanin and elastase levels decreased in the presence of compounds in a dose-dependent manner. We hypothesize that our compounds impaired virulence factor production by inhibiting the *P. aeruginosa* sensing of AI-2 signal molecules derived from *S. aureus* cells.

In conclusion, the molecules studied in this work are very promising in decreasing both *P. aeruginosa* and *S. aureus* virulence; therefore, they provide a potential new strategy for the clinical treatment of multidrug-resistant bacterial infections. Future studies on clinical MDR isolates will be performed to further validate the translational relevance of our findings and to elucidate the exact mechanisms of action and possibly identify the QS factors involved in the AI-2 inhibitory process.

## 4 Materials and Methods

### 4.1 Bacterial Strains and Growth Conditions

The *P. aeruginosa* reference strain PA01 (kind gift from professor Silvia Buroni)^49^ and *S. aureus* reference strain SH1000 wild-type and its derivative Δ*spa* strain (laboratory collection)^50,51^ were cultured in tryptic soy broth (TSB) at 37 °C and 180 rpm for epithelial cell adhesion assays or Luria–Bertani (LB) medium at 37 °C and 150 rpm for pyocyanin or elastase tests. For ELISAs involving bacterial immobilization, cultures were centrifuged to harvest the bacteria, which were then washed and resuspended in phosphate-buffered saline (PBS, NaCl 130 mM, Na_2_HPO_4_ 7 mM, NaH_2_PO_4_ 3 mM, pH 7.4) in equal concentrations (optical density at 600 nm = 1.0).

### 4.2 Compounds, Reagents, and Antibodies

Skim milk and bovine serum albumin (BSA) were obtained from Sigma-Aldrich. Compounds PV-DPD-6, PV-DPD-16, and PV-DPD-19 were previously synthesized as described in Milli et al.^38^ and were dissolved in DMSO. The batch purity of compounds met the typical criteria of >95%.

Primary polyclonal antibodies were obtained by routinely immunizing mice with purified bacterial proteins as antigens, as previously reported.^52^ The study complies with the ARRIVE guidelines (https://arriveguidelines.org), with antibody production procedures performed in accordance with all relevant regulations. All experimental protocols were conducted upon approval by the University of Pavia’s ethical review board.

Horseradish peroxidase (HRP)-conjugated secondary antibodies were purchased from Dako Cytomation (Glostrup, Denmark). OPD tablets (*o*-phenylenediamine dihydrochloride) were purchased from ThermoScientific (Rockford, IL, USA).

### 4.3 Mammalian Cell Culture and Epithelial Cell Adhesion Assays

A549 cells were routinely maintained in 75 cm^2^ flasks using Dulbecco’s Modified Eagle’s Medium (DMEM) supplemented with 10% fetal bovine serum (FBS) at 37 °C under 5% CO_2_. For adhesion assays, cells were seeded at a density of 1.5 × 10^5^ cells per well in 24-well tissue culture plates and cultured in DMEM without antibiotics for 24 h. Bacteria were cultured overnight in a TSB medium, either with or without the compounds, and then added to the cell monolayers (multiplicity of infection, MOI, of 10) following washing and resuspension in PBS. After 1 h of incubation, nonadherent bacteria were removed by washing the cells three times with PBS, after which the cells were lysed and plated in serial dilutions to enumerate cell-associated bacteria. Adhesion for each strain (%) was calculated as (CFUs recovered from the plate ÷ CFUs of the initial inoculum) × 100 and normalized to the untreated strain, which was defined as 100%.

### 4.4 ELISA

ELISA was used to evaluate the expression of adhesins of interest after bacterial growth in the presence or absence of compounds. Briefly, cells cultured overnight in TSB were collected by centrifugation, washed, and resuspended in PBS (OD_600_ = 1). Aliquots (100 μL) were immobilized onto microtiter plate wells by incubation at 37 °C overnight. Wells were then washed three times with PBST (PBS containing 0.5% [v/v] Tween 20) and blocked for 1 h at 22 °C with BSA 2% (v/v) in PBS. Surface expression of MSCRAMMs on immobilized bacteria was assessed by treating the wells for 1 h with mouse polyclonal antibodies specific to each adhesin (1:1000 in 1% [v/v] BSA), followed by a 45 min incubation with HRP-conjugated antimouse IgG secondary antibody (1:1000 in 1% [v/v] BSA). OPD substrate was added following washing, and absorbance was recorded at 490 nm using a Bio-Rad ELISA plate reader.

### 4.5 Western Blot Analysis

To assess the secretion of SpA and Hla in *S. aureus* culture supernatants, bacterial cultures were cultured overnight with or without compounds. After centrifugation, the supernatants were filtered through 0.22 μm membranes and concentrated approximately 10-fold at 4 °C using Amicon Ultra centrifugal devices (MWCO 10 kDa). Five microliters of each sample were loaded onto a 12.5% SDS-PAGE gel and transferred to a PVDF membrane (Bio-Rad). The membrane was blocked overnight at 4 °C in 5% (w/v) skim milk in PBST, followed by incubation for 1 h at 22 °C with 1 μg/mL rabbit anti-SpA or anti-Hla antibody in 2% (w/v) skim milk. After several washings with PBST, the membrane was treated with an HRP-conjugated rabbit antimouse IgG (1:1000) in 2% (w/v) skim milk for 45 min at 22 °C and blots were subsequently developed using the Westar Supernova detection kit (Cyanagen srl, Bologna, Italy). Images of the bands were captured using an ImageQuant LAS 4000 minibiomolecular imager (GE Healthcare). Signal intensities were quantified with ImageJ and plotted with GraphPad Prism.

### 4.6 Pyocyanin Quantification

Pyocyanin was quantified as previously published (18). Briefly, *P. aeruginosa* PAO1 and *S. aureus* SH1000 were grown in LB medium at 37 °C and 150 rpm until the exponential phase of growth (OD = 0.4–0.6). The culture mix was diluted 10-fold with fresh PB medium following mixing in equal proportions. Five mL of each bacterial culture with and without the test compounds (20 and 40 μM for each compound) were incubated at 37 °C and 150 rpm for 16 h. The culture was centrifuged at 4000 rpm for 10 min, and the supernatant was isolated from the bacterial pellet in order to extract pyocyanin with 3 mL of chloroform. Finally, 1 mL of 0.2 M HCl was added to the chloroform layer and thoroughly mixed, and after centrifugation at 4000 rpm for 10 min, the upper phase was collected. The absorbance was measured at 520 nm, and the pyocyanin levels were represented as % of the control (untreated coculture), set as 100%.

### 4.7 Elastase Quantification

For elastase measurement, *P. aeruginosa* PAO1 and *S. aureus* SH1000 were cultured in a PTSB medium at 37 °C with shaking at 150 rpm until reaching the exponential growth phase. Cultures were then mixed in equal proportions, divided into experimental and control groups, and incubated with or without the compounds at 37 °C and 150 rpm for 18 h. Following incubation, samples were centrifuged at 9,590*g* for 5 min, and the supernatants were collected. These supernatants were filtered through 0.22 μm pore-size filters, and 200 μL of the filtrate was combined with 1 mL of Elastin–Congo red reaction solution (20 mg/mL Elastin–Congo red, 0.1 M Tris–HCl, pH 7.2, 1 mM CaCl_2_) and incubated at 37 °C with shaking at 150 rpm for 18 h. The reaction was stopped by adding 100 μL of 0.12 M EDTA, followed by 5 min on ice and centrifugation at 9,590*g* for 10 min. The absorbance of the collected supernatants was measured at 495 nm. Elastase activity was expressed as a percentage of the untreated coculture, which was defined as 100%.

### 4.8 Measurement of *S. aureus* AI-2 Activity through a *Vibrio harveyi* Bioluminescence Assay

Analysis of the AI-2 activity of culture supernatants was carried out as previously reported.^53^ Briefly, the *V. harveyi* BAA-1117 strain (ATCC) was cultured in Autoinducer Bioassay (AB) medium at 30 °C with shaking (100 rpm). The AB medium was prepared with 0.3 M NaCl, 0.05 M MgSO_4_, and 2% casamino acids, and the pH was adjusted to 7.5 using 1 M NaOH. Following autoclaving at 121 °C, the following sterile supplements were added per 1 L of medium: 10 mL of 1 M potassium phosphate (pH 7.0), 10 mL of a 0.1 M l-arginine solution, and 10 mL of glycerol. HEPES buffer 25 mM, pH 7.8, was added to the AB medium to counteract changes in bioluminescence due to the addition of acidic culture supernatants. *S. aureus* was cultured overnight in the TSB medium supplemented with 0.5% glucose, at 37 °C, in the presence or absence of PV-DPD-6, PV-DPD-16, or PV-DPD-19 compound (2 mM). Overnight bacterial cultures were filter-sterilized, and cell-free supernatants (CFS) were diluted 1:10 in the modified AB medium containing *V. harveyi* cells (diluted 1:5000 from the overnight culture) to give a final volume of 200 μL. The plate was incubated at 30 °C with shaking at 100 rpm. Bioluminescence (OD_490_ nm) and cell density (OD_600_ nm) were measured with a ClarioStar reader. Bioluminescence readings were normalized to cell density, and the AI-2 activity of bacteria exposed to each CFS was expressed as a percentage relative to the control (bacterial cells resuspended in CFS from *S. aureus* grown without compounds), which was set at 100%.

### 4.9 Compounds Cytotoxicity In Vitro and In Vivo

The cytotoxicity of compounds was determined for the A549 cell line. Cells previously cultured in DMEM supplemented with 10% fetal bovine serum were seeded in 96-multiwell sterile plates (10^5^ cells/well) and allowed to reach confluency. After 24 h of incubation at 37 °C and 5% CO_2_, 2-fold serial dilutions of each compound were added in 200 μL cell culture medium per well and incubated at 37 °C in 5% CO_2_ for 24 h. Postincubation, 30 μL of 0.01% resazurin (Sigma-Aldrich) was added per well and the plate was incubated at 37 °C for a further 4 hours. Pink resorufin obtained by reduction of blue resazurin by metabolically active cells was quantified by reading resorufin fluorescence (ex: 520 nm, em: 580 nm) using a ClarioSTAR microplate reader.

Larvae were obtained from a local supplier in Pavia and organized into Petri dishes with a minimum of 10 larvae per group. The experiment was carried out by inoculating different concentrations of each compound (up to 200 μM) dissolved in DMSO with an injection volume of 10 μL per larva (between 0.2 and 0.3 g in weight), into the front proleg using a microsyringe (Hamilton Ltd.). Three controls were employed for the assay: (i) untreated larvae maintained under the same conditions as the treated larvae; (ii) larvae injected with 10 μL of PBS solution; (iii) larvae treated with 10 μL of a sterile DMSO solution. Treated larvae were placed in sterile Petri dishes and incubated at room temperature (22 °C) for up to 3 days. Larval viability was recorded at 24, 48, and 72 h postincubation, analyzing the complete loss of mobility after physical stimulus using a plastic pipet together with discoloration of the cuticle, suggestive of larval death.

### 4.10 Statistical Methods

Data were analyzed by using Prism 9.1 (GraphPad). Each experiment included at least three biological replicates (three independent experiments). For comparisons involving more than two groups, one-way ANOVA followed by Dunnett’s post hoc test was used. *P* values < 0.05 were considered statistically significant.
